# Universality vs. Cultural Specificity in the Relations Among Emotional Contagion, Emotion Regulation, and Mood State: An Emotion Process Perspective

**DOI:** 10.3389/fpsyg.2019.00186

**Published:** 2019-02-12

**Authors:** Beibei Kuang, Shenli Peng, Xiaochun Xie, Ping Hu

**Affiliations:** ^1^Department of Psychology, Renmin University of China, Beijing, China; ^2^School of Psychology, Northeast Normal University, Changchun, China

**Keywords:** emotional contagion, emotion regulation, mood state, universality, cultural specificity, emotion processing

## Abstract

To investigate the universality and cultural specificity of emotion processing in children from three different ethnic groups (Han, Jingpo, and Dai), we administered three questionnaires, including the emotional contagion scale, emotion regulation scale, and the Chinese mood adjective check list, to 1,362 ethnic Han, Dai, and Jingpo participants (*M*_age_ = 13.78 years). Structural equation modeling was used to examine the universality and cultural specificity in the relations among emotional contagion, emotion regulation, and mood state. The results revealed that emotion regulation mediated the relation between emotional contagion and mood state: positive emotional contagion increased positive mood state and decreased negative mood state by the mediated role of reappraisal, negative emotional contagion decreased positive contagion and increased negative mood state by the inconsistent mediated role of reappraisal; negative contagion increased negative mood state by the mediated role of suppression. We found both universality and cultural specificity in the relations among emotional contagion, emotion regulation, and mood state. Regarding cultural specificity, among Dai and Jingpo participants, negative contagion positively predicted reappraisal, while for Han participants, it did not; Jingpo participants demonstrated a weaker negative relation between reappraisal and negative mood state, and a stronger positive relation between negative contagion and suppression; and Dai participants were the only ethnic group that showed a negative connection between negative contagion and positive mood state. Regarding emotion universality, the three ethnic groups all showed positive relations between negative contagion and negative mood, and between suppression and negative mood; additionally, positive contagion positively predicted positive mood state, mediated by reappraisal. Thus, some emotion processes are universal and others more specific. In this paper, we discuss universal emotion processes and ethnic cultural differences in these emotion processes.

## Introduction

Are emotions universal or culturally specific? The nature of emotion has generated considerable debate (e.g., Adolphs, 2017; Barrett, 2017). Emotion universality has attracted researchers’ attention since the seminal work of Ekman (1999). Emotion universality theories assume that emotions are innate and universal, independently of human’s acknowledgment of them, and that all humans have the capacity to experience and perceive the same core set of emotion categories. Ekman and his colleagues have conducted many representative studies of this view (e.g., Ekman, 1971; Ekman and Friesen, 1971, 1986), leading to the development of the Facial Affect Scoring System. However, this theory has received theoretical (Scarantino, 2012; Celeghin et al., 2017) and experimental (Elfenbein and Ambady, 2002, 2003) challenges. Psychological constructionists claim emotions are “situated conceptualizations" and socially constructed (Barrett, 2009; Lindquist et al., 2012), and emerge when people make meaning out of sensory input from the body and from the world using knowledge of prior experiences. They propose that emotions are realized by basic psychological components that are not emotion-specific, but which are selected for particular emotions on certain occasions. Lindquist et al. (2012) proposed four components of emotion: sensory input (such as raw somatic sensations, motor cues, or affect), prior experiences (such as attribution), emotion words, and executive attention; this model views emotion as a whole process. We also believe that the nature of emotion (universality or cultural specificity) should be explored from a whole process perspective.

The idea that emotion is a whole process is not new and has been clearly illustrated. Researchers have proposed that emotion information should be viewed as a dynamic process (Solomon and Corbit, 1978) that is organized hierarchically (Leventhal, 1984; Brown, 1985; Öhman, 1993). Sonnby-Borgström et al. (2008) reviewed previous studies and concluded that emotion is *a three-layered system*. The first level is assumed to be inherited and biologically prepared, and is either physiological or an automatic motor process, evoked by specific stimuli. At the second level, a schema-driven elevator operates without access to conscious awareness. The third step is at the controlled or emotionally regulated level and activates conscious perception channels. Thus, sensory input (e.g., external stimuli, emotional input which is transformed from others) will not maintain an initial state but will undergo a regulation process. Finally, individual emotions will become stable and form different mood states. The current study focused on the nature (universality or cultural specificity) of emotion processes, including emotional contagion (sensory input), emotion regulation, and their resulting mood states. We will review previous studies of emotional contagion and emotion regulation, and then propose our own hypotheses.

Emotional contagion has been defined as the tendency to automatically mimic and synchronize others’ facial expressions, vocalizations, postures and other bodily states (Hatfield et al., 1993; Prochazkova and Kret, 2017). During the emotional contagion process, individual affect is automatically activated by these sensory inputs, which belong to *the first level of the three-layered system* (Sonnby-Borgström et al., 2008). Theorists claim that emotional contagion is universal and innate, since the phenomenon of emotional contagion has been observed in a number of cultures (for example, American, Finnish, Dutch, Western European, and Japanese), while many researchers have pointed out that culture can enhance or inhibit these processes (Hatfield et al., 2014). For example, Ilies et al. (2007) found that team members who were more collectivistic were more susceptible to affective influences from other team members compared to those who were more individualistic. The nature of emotional contagion is not yet clear.

Emotion regulation refers to the process by which individuals influence their emotions, when they have them, and how they experience and express these emotions (Gross, 1998), and belongs *to the third level of the three-layered system* (Sonnby-Borgström et al., 2008). Cognitive reappraisal and expression suppression are believed to be widely used and particularly potent emotion regulation strategies. Cognitive reappraisal involves cognitively transforming one’s perception of the situation so as to alter its emotional impact (Lazarus and Alfert, 1964; Gross et al., 2006). Expression suppression involves inhibiting ongoing emotion-expressive behavior (Gross et al., 2006). Both universality and cultural specificity have been found in the use and impact of these two strategies. For example, Haga et al. (2009) found that Americans used more expression suppression than did Norwegians and Australians, and Australians used more reappraisal compared to Norwegians. Gross and John (2003) found that Americans used less suppression than ethnic minorities, but no differences in reappraisal were found. As for the impact of emotion regulation, Butler et al. (2007) found the use of suppression was not as deleterious for individuals holding Asian values, compared with individuals holding Western-European values, while Roberts et al. (2008) found that culture failed to moderate the effect of emotion suppression. Therefore, emotion regulation can simultaneously be considered from the perspective of universality and specificity.

One important possible reason for the lack of consensus among the results may be the content of the research. Previous research explored emotional contagion and emotion regulation separately and neglected to consider them as an integrated overall process. In this paper, we explore universality and cultural specificity of emotion from the perspective of the emotion process based on the relations among different emotion domains. Since this is exploratory research, we are open to hypotheses about its universality and cultural specificity. Next, we will illustrate the relations between emotional contagion, emotion regulation, and mood state.

### Development of Hypotheses

Emotional contagion is common in daily life and transfers emotions from senders to observers (Schoenewolf, 1990). Thus, the receivers’ mood state is influenced by that of the sender. Our research here focuses on receivers. The intensity of received emotions is supposed to be influenced by observers’ susceptibility to expressions. In this paper, emotional contagion refers to emotional contagion susceptibility. Studies have found that emotional contagion played a substantial role in affecting receivers’ mood states among infants (Waters et al., 2017), adolescent siblings (Serra Poirier et al., 2017), and adults (Wu et al., 2014; Volante et al., 2016). More often, emotional contagion has been found to play a key role in industrial and organizational areas among adults. For example, the display of a positive emotion by employees is found to be positively related to customers’ positive affect (Pugh, 2001). Moreover, researchers found various other forms of emotional contagion. For example, Facebook users who received fewer positive expressions produced fewer positive posts and more negative posts (Fan et al., 2016; Kramer et al., 2014). Therefore, emotional contagion can influence individuals’ current mood.

Hypothesis 1: Emotional contagion will predict individual mood states.
Hypothesis 1a: Positive emotional contagion will reinforce a positive mood and reduce a negative mood.
Hypothesis 1b: Negative emotional contagion will reinforce a negative mood and reduce a positive mood.

To get along with others, individuals must manage and regulate their own emotions. To explain how individuals regulate mood states, Gross (1998) proposed a process model of emotion regulation that includes five regulation sets and emphasized: (a) the regulation targets include not only positive but also negative emotions; (b) the direction of regulation includes increasing, decreasing, and maintaining the current mood; and that (c) widely used and particular potent forms during the process are cognitive reappraisal and expression suppression. Importantly, different regulation strategies were found to have different impacts. For example, Gross and John (2003) found that reappraisal increased positive emotional experiences and decreased negative emotional experiences, and that suppression decreased positive emotions. Compas et al. (2017) found that emotional suppression was significantly positively associated with internalizing symptoms during childhood and adolescence. In this regard, meta-experiences of mood can be conceptualized as the result of a regulatory system (Mayer and Gaschke, 1988). Therefore, we propose that mood state can be predicted by emotion regulation.

Hypothesis 2: Emotion regulation will predict mood state.
Hypothesis 2a: Cognitive reappraisal will positively predict positive mood and negatively predict negative mood.
Hypothesis 2b: Suppression will negatively predict positive mood and positively predict negative mood.

According to the three-layered emotion system (Sonnby-Borgström et al., 2008), emotional contagion, operating automatically, evoked by others’ affect, is the first level of the three-layered system; emotion regulation is the middle process following emotional contagion; and mood state (the end product) is the result of emotional contagion and emotion regulation (a microprocess in time) (Brown, 1985).

Experimental evidence that emotional contagion is related to emotion regulation has been found. Sonnby-Borgström et al. (2008) found gender differences in verbally reported emotional contagion. They proposed that these identified differences could be interpreted by the emotion regulation differences between men and women. Two additional studies that explored the relation between emotional contagion and emotion regulation were conducted by Papousek et al. (2008, 2011). They found (Papousek et al., 2008) that when participants viewed a sad film, those who were good at emotion regulation and weak at emotion perception experienced the weakest impact of emotional contagion (the film). In their other study (Papousek et al., 2011), they found that individuals with higher scores on emotion regulation showed better recovery of prefrontal cortical asymmetry changes, which resulted from emotional contagion. It is possible that emotional contagion can influence individuals’ mood states through emotion regulation. However, Papousek et al. (2008, 2011) neglected to directly measure contagion, and only conducted their experiment employing a contagion paradigm in a lab environment. The current investigation explores the relations among emotional contagion, emotion regulation, and mood state directly, employing questionnaires.

Hypothesis 3: Emotional contagion susceptibility will affect mood state directly and indirectly through emotion regulation.
Hypothesis 3a: Positive contagion by reappraisal will increase positive mood and decrease negative mood.
Hypothesis 3b: Positive contagion by suppression will decrease positive mood and increase negative mood.
Hypothesis 3c: Negative contagion by reappraisal will increase positive mood and decrease negative mood.
Hypothesis 3d: Negative contagion by suppression will decrease positive mood and increase negative mood.

### The Present Study

China consists of more than 56 ethnic groups, of which the Han people are the largest one. Each ethnic group typically possesses their own language, traditions, customs, etc. For example, the Dai people dance and express happiness after the death of a relative, the Jingpo people dance to chant the achievements of the dead and reduce the pain of the dead person’s relatives, while Hans cry and express sadness. Exploring differences and common ground in the emotion processes among different groups aids mutual understanding. In this paper, we explore the universality and cultural specificity of emotion processes among three ethnic groups in China.

First, we examined the relation between emotional contagion and mood state among children. A typical limitation of previous studies has been that most of the research about emotional contagion has focused on adults (e.g., Kramer et al., 2014; Wu et al., 2014; Fan et al., 2016; Volante et al., 2016). According to computer searches of PsycInfo, 417 of 690 studies focused on adults, 53 studies focused on adolescents, and 54 studies focused on children. Emotional contagion provides an emotional environment for individuals. Children are more easily influenced by their surroundings. Therefore, research on children may advance our understanding of the relation between emotional contagion and mood state.

Second, our study extends previous research by examining the role of emotion regulation between emotional contagion and mood state. Previous research has focused mainly on the separate effects of emotional contagion (e.g., Serra Poirier et al., 2017) and regulation on mood state (e.g., Zimmermann and Iwanski, 2014). According to computer searches of PsycInfo, 44 of 822 studies involved emotional contagion and regulation simultaneously. Exploration of the links among emotional contagion, emotion regulation, and mood state can facilitate our understanding of the emotion process.

Third, this research explores emotion universality and ethnic differences based on the relations among emotional contagion, emotion regulation, and mood state.

## Materials and Methods

### Participants and Procedure

A total of 1,353 participants took part in our research. There was a total of 76 (6%) missing responses. Data were analyzed from 1,277 participants who had middle or higher scores in Chinese language (i.e., Mandarin) reading comprehension, including 510 males and 695 females (72 participants were unspecified). The average age of the participants was 13.78 ± 2.45 years with a range of 9–18. The sample consisted of three ethnic groups in China: 460 (36%) Han (*M*_age_ = 13.69, *SD* = 2.61), 357 (28%) Dai (*M*_age_ = 14.02, *SD* = 2.10), and 384 (30%) Jingpo (*M*_age_ = 13.67, *SD* = 2.54) participants. There were no significant age differences between the three ethnic groups [*F*_(2,1166)_ = 2.415, *P* = 0.09].

We developed a questionnaire that consisted of scales assessing emotional contagion, emotion regulation, and mood state. Participants completed the questionnaire in about 15 min. We explained all the details of our research to the appropriate schools’ personnel and to each participant, who were informed that they could quit and withdraw from the study at any time. We received written informed consent from all participants. This study was approved by the ethics board of the Renmin University of China, as well as by the participating schools.

### Measures

The Chinese version (Wang et al., 2013) of the *Emotional Contagion Scale* (Doherty, 1997) was employed to measure emotional contagion. It has been widely used among Chinese studies. The scale consists of 13 items, including five dimensions: happiness, love, fear, anger, and sadness, with response values ranging from 1 (*never true for me*) to 4 (*always true for me*). The higher the scores, the higher the susceptibility to emotional contagion. To make the measurement suitable for children, we amended the wording “lovers” to “parents” in three items (α = 0.833): “When I look into the eyes of the one I love, my mind is filled with thoughts of romance” was changed to “When I look into the eyes of my parents, my mind is filled with thoughts of warmth.” “I melt when the one I love holds me close” was changed to “I melt when my parents holds me close.” “I sense my body responding when the one I love touches me” was changed to “I sense warmth when my parents touch me.” An exploratory factor analysis (EFA) and confirmatory factor analysis (CFA) were conducted to verify the validity of the measurement. The EFA results revealed two dimensions, positive emotional contagion (α = 0.749) and negative emotional contagion (α = 0.721). In the CFA, the following indices were used to assess goodness of fit: the comparative fit index (CFI), root mean square error of approximation (RMSEA), standardized root mean square residual (SRMR), and the chi-square statistic (χ^2^). Cut-off values (CFI close to 0.95, RMSEA close to 0.06, and SRMR close to 0.08) were selected based on the research recommendations of Hu and Bentler (1999, p. 1). The modeling indexes [χ^2^(66) = 255.472, *p* < 0.0001; CFI = 0.922, SRMR = 0.034, RMSEA = 0.062] were acceptable.

The Chinese version of the *Emotion Regulation Questionnaire for Children and Adolescents* (Chen et al., 2016) was applied to measure emotion regulation. It was developed by Gullone and Taffe (2012). This questionnaire includes a reappraisal scale (α = 0.841) and a suppression scale (α = 0.763) and consists of 10 items, such as “I control my emotions by changing the way I think about them” (reappraisal) and “I control my feelings by not showing them” (suppression), ranging from 1 (*strongly disagree*) to 7 (*strongly agree*). A CFA was conducted and its modeling indexes [χ^2^(64) = 170.699, *p* < 0.001; CFI = 0.966, SRMR = 0.034, RMSEA = 0.037] showed good fit.

Mood state was measured by the Chinese mood adjective checklist developed by Zhong and Qian (2005). It includes thirty items (e.g., distressed, happy) that are scored from 1 (*disagree very much*) to 5 (*agree very much*). Twenty of these items were chosen to make up the briefer children’s version. An EFA and CFA were conducted to verify the validity of the measurement. Our EFA revealed four dimensions: fidget (α = 0.806), happy and excited (α = 0.924), pain and sadness (α = 0.897), and anger and hate (α = 0.903), which were the same as those identified by the authors. The modeling indexes of the CFA [χ^2^(116) = 425.421, *p* < 0.001; CFI = 0.943, SRMR = 0.018, RMSEA = 0.048] were acceptable. To obtain a relatively simplified model, we included a positive mood dimension including “happy” and “excited” (α = 0.933) and a negative mood dimension (α = 0.883) that included the combined score of the other three dimensions.

### Data Analysis

We first explored the relations among three key research variables, by conducting mediation model analysis with emotional contagion as the independent variable, mood state as the dependent variable, and emotion regulation as the mediation variable. Then we tested the universality and specificity of these relations, by comparing the mediation model among the three ethnic groups. Our analyses were performed with Mplus version 7.4 (Muthén and Muthén, 1998/2015) using the maximum likelihood estimator to fit our models. Missing data were handled according to the default options of Mplus. Model fit was determined via the CFI (CFI: ≥0.95 indicates a good fit and between 0.95 and 0.90 indicates an acceptable fit) (Hu and Bentler, 1999) and the RMSEA (RMSEA: ≤0.05 indicates a close approximate fit, between 0.05 and 0.08 indicates a reasonable error of approximation and ≥1.00 indicates a poor fit) (Browne and Cudeck, 1993). We used 1,000 bootstrap resamples with replacement and bias-corrected 95% confidence intervals (95% CI; Preacher and Hayes, 2004) to estimate the significance of the indirect effects.

## Results

### Descriptive Results

The descriptive results are displayed in Table 1. The ethnic differences in key research variables were assessed with a one-way ANOVA. Significant main effects were found in negative contagion [*F*_(2,1146)_ = 3.161, *p* = 0.043, $\eta_{p}^{2}$ = 0.01] and reappraisal [*F*_(2,1144)_ = 4.822, *p* = 0.008, $\eta_{p}^{2}$ = 0.01]. Next, we conducted *post hoc* analysis: Dai participants showed lower negative contagion (*M* = 3.33) than Hans (*M* = 3.45) and Jingpos (*M* = 3.45), and showed lower reappraisal (*M* = 4.53) than Hans (*M* = 4.77) and Jingpos (*M* = 4.71). No significant differences in suppression and mood state (positive and negative mood state) were found [*F*_(2,1146)_ = 0.852, *p* = 0.427, $\eta_{p}^{2}$ = 0.00; *F*_(2,1146)_ = 0.935, *p* = 0.393, $\eta_{p}^{2}$ = 0.00; *F*_(2,1146)_ = 0.270, *p* = 0.763, $\eta_{p}^{2}$ = 0.00].

**Table 1:** Correlations among the key variables.

Variables	1	2	3	4	5	6
(1) Positive contagion	–					
(2) Negative contagion	0.56^∗∗^	–				
(3) Reappraisal	0.40^∗∗^	0.34^∗∗^	–			
(4) Suppression	0.12^∗∗^	0.19^∗∗^	0.52^∗∗^	–		
(5) Positive mood	0.25^∗∗^	0.09^∗∗^	0.21^∗∗^	0.05	–	
(6) Negative mood	−0.11^∗∗^	0.01	−0.08^∗∗^	0.15^∗∗^	0.11^∗∗^	–
*M (SD)_Total_*	3.91 (0.81)	3.41 (0.75)	4.68 (1.10)	4.07 (1.07)	3.57 (1.11)	2.31 (0.81)
*M (SD)_Han_*	3.95 (0.82)	3.45 (0.75)	4.77 (1.07)	4.06 (1.07)	3.60 (1.11)	2.33 (0.89)
*M (SD)_Dai_*	3.84 (0.77)	3.33 (0.71)	4.53 (1.08)	4.01 (1.05)	3.51 (1.09)	2.31 (0.75)
*M (SD)_Jingpo_*	3.92 (0.84)	3.45 (0.77)	4.71 (1.13)	4.12 (1.09)	3.61 (1.12)	2.29 (0.77)

*^∗∗^P < 0.001, ^∗^P < 0.005.*

Pearson’s product-moment correlations were computed among all the research variables. Table 1 shows that the key variables in this study were generally significantly correlated with each other with two exceptions—the relation between negative contagion and negative mood, and the relation between suppression and positive mood.

### Mediation Model Analysis

**FIGURE 1:**
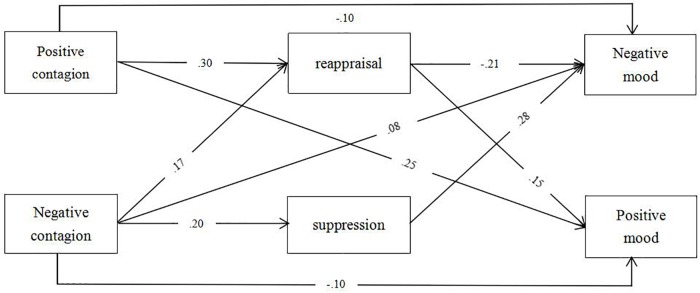
Mediation model of emotion regulation between emotional contagion and mood state. The results shown are from our restricted model (standardized estimates). All of the reported coefficients were significant at *p* < 0.05.

We next conducted path analysis based on the correlation results. Our structural equation model, which included positive emotion, negative emotion, reappraisal, suppression, positive mood, and negative mood, was tested employing Mplus with emotional contagion as the independent variable, emotion regulation as the mediator, and mood state as the dependent variable. After removing some paths step-by-step that were not significant (two-sided: *p* > 0.05), the modeling indexes of our mediation model [χ^2^(2) = 2.942, *p* = 0.230; CFI = 0.999, SRMR = 0.008, RMSEA = 0.019] showed good fit. The mediating role of emotion regulation between emotional contagion and mood state was found. Significant paths of the mediation model and the standard estimates for the significant paths are displayed in Figure 1. The direct and indirect effects are reported below.

#### Direct Effects

We report direct effects of independent variables on dependent variables, independent variables on mediators, and mediators on outcomes, respectively.

First, the direct effects of the independent variables (emotional contagion) on dependent variables (mood state) were estimated. Positive contagion (β = 0.25, *p* < 0.001) and negative contagion (β = −0.10, *p* = 0.006) significantly predicted positive mood, and positive contagion (β = −0.10, *p* = 0.012) and negative contagion (β = 0.08, *p* = 0.019) significantly predicted negative mood. The direct effect of positive contagion on positive mood was the largest among four direct effects, explaining 25% of the variance in positive mood. This result indicated that emotional contagion influenced mood state: positive contagion increased positive mood and decreased negative mood; negative contagion increased negative mood and decreased positive mood. Therefore, Hypothesis 1 was supported.

Second, the direct effect of the independent variables (emotional contagion) on mediators (emotion regulation) were estimated. Both positive contagion (β = 0.30, *p* < 0.001) and negative contagion (β = 0.17, *p* < 0.001) were significantly positive predictors of reappraisal. However, only negative contagion (β = 0.20, *p* < 0.001) was a significant positive predictor of suppression. These results indicated that individuals with higher positive contagion tended to use reappraisal, and individuals with higher negative contagion tended to use suppression and reappraisal simultaneously.

Third, the direct effects between the mediators (emotion regulation) and outcomes (mood state) were also estimated. For positive mood, reappraisal (β = 0.15, *p* < 0.001) was a significant positive predictor; however, suppression was not a significant predictor. For negative mood, reappraisal (β = −0.21, *p* < 0.001) was a significant negative predictor and suppression (β = 0.28, *p* < 0.001) was a significant positive predictor. These results indicated that reappraisal decreased individuals’ negative mood states and increased their positive mood states, and that suppression enhanced individuals’ negative mood states. Therefore, Hypothesis 2a was supported, and Hypothesis 2b was partially supported.

#### Indirect Effects

We found a mediating role of reappraisal between emotional contagion and mood state, and a mediating role of suppression between negative contagion and negative mood. Indirect effects of reappraisal and suppression are each reported and are summarized in Table 2. For the indirect effects, 95% bootstrap confidence intervals (CI) without “zero” indicated a significant mediation effect.

**Table 2:** Effect size of indirect paths.

	Effect	95%CI	Ratio of indirect effect to direct effect
Positive contagion – reappraisal – positive mood	0.046	0.028, 0.065	16
Positive contagion – reappraisal – negative mood	−0.065	−0.087, −0.045	40
Negative contagion – reappraisal – positive mood	0.026	0.014, 0.040	27
Negative contagion – negative mood	0.020	−0.001, 0.039	
*Negative contagion – reappraisal – negative mood*	−0.037	−0.055, −0.022	46
*Negative contagion – suppression – negative mood*	0.057	0.039, 0.077	71

The standardized indirect effect of reappraisal between positive contagion and positive mood was 0.046, the CI was [0.028, 0.065], and the ratio of the indirect effect to the total effect was 16%. The standardized indirect effect of reappraisal between positive contagion and negative mood was −0.065, the CI was [−0.087, −0.045], and the ratio of the indirect effect to the total effect was 40%. The standardized indirect effect of reappraisal between negative contagion and positive mood was 0.026, the CI was [0.014, 0.040], and the ratio of the indirect effect to direct effect was 27%. The total standardized indirect effect between negative contagion and negative mood was 0.020 and the CI was [−0.001, 0.039], including two opposite indirect effects. Specifically, *negative contagion predicted negative mood via the weakened effect of reappraisal*, where the standardized indirect effect was −0.037, the CI was [−0.055, −0.022], the ratio of indirect effects to direct effects was 46%, *and via the enhanced effect of suppression*, where the standardized indirect effect was 0.057, CI was [0.039, 0.077] and the ratio of indirect effects to direct effects was 71%. Thus, emotional contagion increased positive mood by the positive mediating role of reappraisal and decreased negative mood by the negative mediating role of reappraisal. Therefore, Hypotheses 3a and 3c were supported. Suppression played a positive mediating role between negative contagion and negative mood. Therefore, Hypothesis 3b was not supported, and 3d was partially supported.

Importantly, for the role of reappraisal, the direct and indirect effects between negative contagion and mood state were in opposite directions. In other words, negative contagion decreased positive mood directly, while it increased positive mood indirectly via reappraisal. Similarly, negative contagion increased negative mood directly, while it decreased negative mood indirectly via reappraisal. Mood state received the direct destructive effect of negative emotional contagion and the indirect protective effect of reappraisal. However, negative contagion inhibited positive mood because the total effect was negative, while it promoted negative mood because the total effect was positive.

In summary, positive contagion benefited current mood, while negative contagion disadvantaged current mood. Reappraisal played a protective role between emotional contagion and mood states, while suppression played a destructive role between negative contagion and negative mood. Positive contagion underwent reappraisal and benefited current mood. Negative contagion underwent not only reappraisal but also suppression. However, these two strategies had opposite indirect effects. That is, people with higher negative contagion felt more negative by suppression, and less negative by reappraisal. Thus, emotional contagion undergoes an adaptation course-emotion regulation. The end product is seen as a result of a microprocess in time (Brown, 1985).

#### Ethnic Differences in the Mediation Model

**FIGURE 2:**
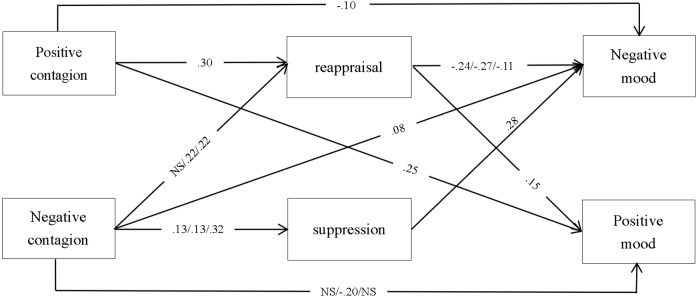
Ethnic differences in emotion processes including emotional contagion, emotion regulation, and mood state. The results shown refer to those from our restricted model (standardized estimates). When two coefficients are presented, the upper coefficient represents estimates of our ethnic Han participants, the middle coefficient represents estimates of our ethnic Dai participants, and the lower coefficient represents estimates of our ethnic Jingpo participants. All of the reported coefficients were significant at *P* < 0.05. NS, not significant.

Next, we estimated the differences in the mediating role of emotion regulation between emotional contagion and mood state among the three ethnic groups. Significant paths of ethnic differences in the mediation model and the standard estimates for the significant paths are displayed in Figure 2.

When we constrained the estimates in our mediation model to be equal for the Han, Dai, and Jingpo participants, there was a significant drop in the model fit [Δχ^2^(20) = 34.26, *p* < 0.05] compared with a freely estimated model. This indicated there were differences among the three ethnic groups. Follow-up analyses indicated various ethnic differences in residual error correlations, in the links between some key variables, and in the four path estimates. After these calculations, we freed these paths one-by-one, beginning each time, from the greatest difference path. When the coefficients for the four individual paths (as well as the significantly different residual error correlations and links among some key variables) were allowed to vary freely among the three ethnic groups (i.e., when these coefficients were freely estimated), the result was no longer a significant drop in fit [Δχ^2^(18) = 8.84, *p* > 0.05] compared with the totally unconstrained model. This indicated the four individual paths were different among the three ethnic groups.

Our results revealed that negative contagion significantly and negatively predicted positive mood for our Dai participants but not for the other two ethnic groups. In addition, negative contagion significantly and positively predicted reappraisal for our Dai and Jingpo participants but it was not significant for the Han participants. Finally, Jingpo participants showed a stronger positive association between negative contagion and suppression and a weaker negative connection between reappraisal and negative mood, compared with the other two ethnic groups. Hence, both universality and ethnic specificity were found based on an emotion process perspective.

In summary, Dai and Jingpo participants’ results showed that negative contagion influenced negative mood simultaneously via both reappraisal and suppression (i.e., the indirect effect of reappraisal was negative and the indirect effect of suppression was positive). Our Han participants’ results showed that negative contagion influenced negative mood positively, although via suppression only.

## Discussion

This paper aimed to explore universality and cultural specificity in the relations among emotional contagion, emotion regulation, and mood stage. As we predicted, Hypothesis 1 was supported (emotional contagion could predict mood state), Hypothesis 2 was partially supported (emotion regulation influenced mood state, except that suppression failed to affect positive mood state), and Hypothesis 3 was partially supported (emotional contagion affected mood state via emotion regulation, except that positive contagion failed to affect mood state via suppression and negative contagion failed to affect positive mood via suppression). Thus, emotion is a whole process including emotional contagion, emotion regulation, and mood state. We also found ethnic differences in the relations between negative emotional contagion and emotion regulation, between negative emotional contagion and positive mood, and between reappraisal and negative mood. It reveals that some emotion processes are universal and others more specific. Matsumoto (1993) suggested that ethnic differences should be interpreted as manifestations of differences in psychological culture, not ethnicity. Ethnicity is defined most often by biological determinants. However, culture must be defined by sociopsychological factors, such as the shared system of beliefs, attitudes, values, and behaviors. Therefore, emotion process can be explained by universality and cultural specificity simultaneously. The universality and cultural specificity of emotion process is discussed based on the relations among emotional contagion, emotion regulation, and mood state.

### The Influence of Emotional Contagion on Mood State

The results of our research showed that positive emotional contagion was positively connected with positive mood state, and negatively connected with negative mood state. Negative emotional contagion was positively connected with negative mood state, and negatively connected with positive mood state. Emotional contagion did influence individual mood state, which is consistent with experimental and social media results (e.g., Ferrara and Yang, 2015; Deng and Hu, 2018). Our results revealed that the contribution of positive contagion to positive mood (β = 0.25) was larger than the contribution of negative contagion to negative mood (β = 0.08). This means that positive expressional information is more likely to be accepted, while negative contagion might undergo more emotion regulation, for example, via suppression (which will be discussed later). This is a possible reason that many experimental studies failed to find consistent results of emotional contagion of negative expression stimuli (Hess and Fischer, 2013). Interestingly, the contribution of positive contagion to negative mood (β = −0.10) was the same as the contribution of negative contagion to positive mood (β = −0.10). In this regard, emotional contagion influenced not only homogenous but also opposite mood states. These results indicate that there are amplification and decay processes from emotional contagion to the mood state.

When we consider the processes among the three different ethnic groups, only Dai participants’ negative emotional contagion decreased positive mood. A possible reason is that Dai people value harmonious relations highly. Previous research (Zhang, 2010) exploring the language structures of different ethnic groups living in China, found that Dai people placed a high value on family and marital relations. After comparing common psychological characteristics of several ethnic groups, Xiong (1994) also found that Dais valued community and harmonious relations. Thus, negative emotional expression from others is more important for Dai people.

### Mediating Roles of Emotion Regulation

Our research indicated that individuals feel more positive and less negative via the mediating role of reappraisal. As for the role of suppression, we only found its mediating role between negative contagion and negative mood. Previous studies have found that results of emotional contagion were influenced by emotion regulation (Papousek et al., 2008, 2011). However, they conducted their experiments using an emotional contagion paradigm without testing emotional contagion directly, and without distinguishing between two different emotion regulation strategies. We extended this and found that people with higher positive contagion susceptibility tended to experience a reappraisal process rather than a suppression process, while people with higher negative contagion susceptibility tended to both reappraise and suppress. Although individuals show negative biases that serve evolutionary and developmental functions (Vaish et al., 2008), in the attention stage, they display a positive bias in the regulation stage (allowing more positive information to pass), as our findings showed.

Positive emotional contagion was positively related to reappraisal, indicating that individuals with higher susceptibility to positive emotional expression (i.e., higher positive contagion) tended to use more reappraisal. Although numerous studies have focused on negative emotion regulation, people do regulate positive emotional information (Tugade and Fredrickson, 2007), by increasing or maintaining emotions. A possible reason is that positive emotions bring people benefits and well-being. According to the broaden-and-build theory (Fredrickson, 2001), positive emotions serve to broaden personal resources, ranging from physical and intellectual resources to social and psychological resources. When people are in a positive state, they will take measures to maintain it (Wegener and Petty, 1994). This trend existed among participants of all three ethnic groups in our study.

Negative contagion was positively related to reappraisal, indicating that individuals with higher susceptibility to negative emotional expression (i.e., higher negative contagion) also tended to use reappraisal. Individuals pay more attention to recognizing negative emotions because they are believed to include more valuable information for human survival, while people tend to decrease negative emotions by reappraisal. This might be because negative emotions expose people to depression and are related to many clinical disorders—for example, anxiety and mood disorders. According to Gross and John (2003), reappraisers negotiate stressful situations by taking an optimistic attitude, reinterpreting what they find stressful, and making active efforts to repair bad moods. We failed to find significant connection between negative contagion and reappraisal for Hans. A possible reason is that Han families are deeply influenced by Confucianism (Zhu, 2015). Confucianism provides a mature “regulation system,” telling people what level their emotions should be kept at (e.g., women should smile without exposing their teeth) and what rules people should obey when they display their emotions (i.e., they are encouraged to suppress them), rather than how to negotiate stressful situations (i.e., use reappraisal) (Zhou, 2007).

Negative emotional contagion positively predicted suppression among people of all three ethnic groups. One possible reason is that expression of negative emotions does harm to relations and creates conflict. People tend to maintain a harmonious relation with others by suppressing their own emotions, rather than creating disharmony. We found stronger connection between negative contagion and suppression among Jingpo participants. A possible reason is that they value social order more. Just as Matsumoto et al. (2008) claimed, in a culture that emphasizes the maintenance of social order, people are more likely to suppress themselves. Jingpo people live in remote areas of China and have great respect for God and the family order. Jingpo people consist of several subgroups, who intermarry each other and speak their own native languages after marriage. Their children follow the father’s culture, and speak to their father in his language, and to their mother in her language. This is because the father’s status is higher than the mother’s, which is part of their traditional family order.

Our findings revealed that reappraisal positively predicted positive mood state and negatively predicted negative mood state, consistent with Gross and John (2003) results. Moreover, reappraisal was also found to increase positive affect (Nezlek and Kuppens, 2008), without significantly decreasing negative affect. Goldin et al. (2008) found that reappraisal decreased negative affect. Although previous studies have been inconsistent, reappraisal is generally believed to positively relate to individual affect and behavior.

Our findings also revealed that suppression positively affected negative mood state, but failed to affect positive mood state, which is inconsistent with the results of Gross and John (2003). They (Gross and John, 2003) found suppressors experienced lesser positive emotion, yet experienced greater negative emotion. This might be due to differences between eastern and western cultures. Evidence has been found by Butler et al. (2007) that suppression is not always related to negative consequences, which was mediated by cultural values. Moreover, on a social level, suppression may play a positive role and cultural function in maintaining cultural systems (Matsumoto et al., 2008). Chinese people tend to suppress their own expressions to achieve group harmony, which makes people feel secure. In our study, such results were universal among the three ethnic groups (three subcultural contexts). Thus, cultural specificity or universality is a relative concept, and should be considered in concrete cultural contexts.

## Conclusion and Limitations

Emotion should be viewed as a whole process, which can be explained simultaneously by universality and cultural differences. Emotional contagion, regulation, and mood state are components of the emotion process, meaning that “received emotion” (emotional contagion) undergoes emotion regulation and then forms mood state. Some processes are universal, while some other processes are ethnically specific; therefore, basic emotion theory seems in need of improvement. Future studies should explore whether there are additional opponents during this process, which may differ according to the emotion types and the relations between senders and receivers.

However, our conclusions should be considered with caution because the present study was conducted among children. Emotion processes of adults and children may be different. Another point is that more attention should be paid to the meaning of the results, rather than the results themselves. Results may differ if a study is conducted among other groups, because our findings were based on three ethnic groups in China (which includes 56 ethnic groups). However, the results reveal a new corner of the nature of emotion, meaning that emotion is a process including several subprocesses. Some subprocesses are universal, while other subprocesses are culturally specific.

## Author Contributions

BK and PH developed the study concept and design. BK and SP collected the data. BK and XX analyzed and interpreted the data. BK drafted the manuscript. PH, XX, and SP revised the draft.

## Conflict of Interest Statement

The authors declare that the research was conducted in the absence of any commercial or financial relationships that could be construed as a potential conflict of interest.

## References

[B1] Adolphs R. (2017). Reply to barrett: affective neuroscience needs objective criteria for emotions. *Soc. Cogn. Affect. Neurosci.* 12 32–33. 10.1093/scan/nsw155 27798258 PMC5390709

[B2] Barrett L. F. (2009). The future of psychology: connecting mind to brain. *Perspect. Psychol. Sci.* 4 326–339. 10.1111/j.1745-6924.2009.01134.x 19844601 PMC2763392

[B3] Barrett L. F. (2017). The theory of constructed emotion: an active inference account of interoception and categorization. *Soc. Cogn. Affect. Neurosci.* 12 1–23. 10.1093/scan/nsw154 27798257 PMC5390700

[B4] Brown J. W. (1985). Clinical evidence for the concept of levels of action and perception. *J. Neurolinguistics* 1 79–87. 10.1016/S0911-6044(85)80005-1

[B5] Browne M. W. Cudeck R. (1993). Alternative ways of assessing model fit. *Sociol. Methods Res.* 21 230–258. 10.1177/0049124192021002005

[B6] Butler E. A. Lee T. L. Gross J. J. (2007). Emotion regulation and culture: are the social consequences of emotion suppression culture-specific? *Emotion* 7 30–48. 17352561 10.1037/1528-3542.7.1.30

[B7] Celeghin A. Diano M. Bagnis A. Viola M. Tamietto M. (2017). Basic emotions in human neuroscience: neuroimaging and beyond. *Front. Psychol.* 8:1432. 10.3389/fpsyg.2017.01432 28883803 PMC5573709

[B8] Chen L. Liu W. Zhang X. (2016). Chinese version of Emotion regulation questionnaire for children and children (ERQ-CA-C). *Chin. J. Clin. Psychol.* 24 259–263. 10.1002/ijop.12233 26611865

[B9] Compas B. E. Jaser S. S. Bettis A. H. Watson K. H. Gruhn M. A. Dunbar J. P. (2017). Coping, emotion regulation, and psychopathology in childhood and adolescence: a meta-analysis and narrative review. *Psychol. Bull.* 143 939–991. 10.1037/bul0000110 28616996 PMC7310319

[B10] Deng H. Hu P. (2018). Matching your face or appraising the situation: two paths to emotional contagion. *Front. Psychol.* 8:2278. 10.3389/fpsyg.2017.02278 29354087 PMC5758747

[B11] Doherty R. W. (1997). The emotional contagion scale: a measure of individual differences. *J. Nonverbal Behav.* 21 131–154. 10.1023/A:1024956003661

[B12] Ekman P. (1971). Universals and cultural differences in facial expressions of emotion. *Nebr. Symp. Motiv.* 19 207–283.

[B13] Ekman P. (1999). “Basic emotions,” in *Handbook of Cognition and Emotion*, eds Dalgleish T. Power M. (Chichester: John Wiley and Sons), 45–60.

[B14] Ekman P. Friesen W. V. (1971). Constants across cultures in the face and emotion. *J. Pers. Soc. Psychol.* 17 124–129. 10.1037/h0030377 5542557

[B15] Ekman P. Friesen W. V. (1986). A new pan-cultural facial expression of emotion. *Motiv. Emot.* 10 159–168. 10.1007/BF00992253 5773719

[B16] Elfenbein H. A. Ambady N. (2002). On the universality and cultural specificity of emotion recognition: a meta-analysis. *Psychol. Bull.* 128 203–235. 10.1037/0033-2909.128.2.203 11931516

[B17] Elfenbein H. A. Ambady N. (2003). Universals and cultural differences in recognizing emotions. *Curr. Dir. Psychol. Sci.* 12 159–164. 10.1111/1467-8721.01252

[B18] Fan R. Xu K. Zhao J. (2016). Easier contagion and weaker ties make anger spread faster than joy in social media. *arXiv* [Preprint]. arXiv:1608.03656

[B19] Ferrara E. Yang Z. (2015). Measuring emotional contagion in social media. *PLoS One* 10:e0142390. 10.1371/journal.pone.0142390 26544688 PMC4636231

[B20] Fredrickson B. L. (2001). The role of positive emotions in positive psychology: the broaden-and-build theory of positive emotions. *Am. Psychol.* 56 218–226. 10.1037/0003-066X.56.3.218 11315248 PMC3122271

[B21] Goldin P. R. McRae K. Ramel W. Gross J. J. (2008). The neural bases of emotion regulation: reappraisal and suppression of negative emotion. *Biol. Psychiatry* 63 577–586. 10.1016/j.biopsych.2007.05.031 17888411 PMC2483789

[B22] Gross J. J. (1998). The emerging field of emotion regulation: an integrative review. *Rev. Gen. Psychol.* 2 271–299. 10.1037/1089-2680.2.3.271

[B23] Gross J. J. John O. P. (2003). Individual differences in two emotion regulation processes: implications for affect, relations, and well-being. *J. Pers. Soc. Psychol.* 85 348–362. 10.1037/0022-3514.85.2.348 12916575

[B24] Gross J. J. Richards J. M. John O. P. (2006). “Emotion Regulation in Everyday Life,” in *Emotion Regulation in Couples and Families: Pathways to Dysfunction and Health*, eds Snyder D. K. Simpson J. Hughes J. N. (Washington, DC: American Psychological Association), 13–35.

[B25] Gullone E. Taffe J. (2012). The emotion regulation questionnaire for children and children (ERQ–CA): a psychometric evaluation. *Psychol. Assess.* 24 409–417. 10.1037/a0025777 22023559

[B26] Haga S. M. Kraft P. Corby E. K. (2009). Emotion regulation: antecedents and well-being outcomes of cognitive reappraisal and expressive suppression in cross-cultural samples. *J. Happiness Stud.* 10 271–291. 10.1007/s10902-007-9080-3

[B27] Hatfield E. Cacioppo J. T. Rapson R. L. (1993). Emotional contagion. *Curr. Dir. Psychol. Sci.* 2 96–100. 10.1111/1467-8721.ep10770953

[B28] Hatfield E. C. Bensman L. Thornton P. D. Rapson R. L. (2014). New perspectives on emotional contagion: a review of classic and recent research on facial mimicry and contagion. *Interpersona* 8 159–179. 10.5964/ijpr.v8i2.162

[B29] Hess U. Fischer A. (2013). Emotional mimicry as social regulation. *Pers. Soc. Psychol. Rev.* 17 142–157. 10.1177/1088868312472607 23348982

[B30] Hu L. T. Bentler P. M. (1999). Cutoff criteria for fit indexes in covariance structure analysis: conventional criteria versus new alternatives. *Struct. Equ. Modeling* 6 1–55. 10.1080/10705519909540118

[B31] Ilies R. Wagner D. T. Morgeson F. P. (2007). Explaining affective linkages in teams: individual differences in susceptibility to contagion and individualism-collectivism. *J. Appl. Psychol.* 92 1140–1148. 10.1037/0021-9010.92.4.1140 17638471

[B32] Kramer A. D. Guillory J. E. Hancock J. T. (2014). Experimental evidence of massive-scale emotional contagion through social networks. *PNAS* 111 8788–8790. 10.1073/pnas.1320040111 24889601 PMC4066473

[B33] Lazarus R. S. Alfert E. (1964). Short-circuiting of threat by experimentally altering cognitive appraisal. *J. Abnorm. Soc. Psychol.* 69 195–205. 10.1037/h0044635 14213291

[B34] Leventhal H. (1984). “A perceptual-motor theory of emotion,” in *Advances in Experimental Social Psychology* Vol. 17 (Cambridge, MA: Academic Press), 117–182.

[B35] Lindquist K. A. Wager T. D. Kober H. Bliss-Moreau E. Barrett L. F. (2012). The brain basis of emotion: a meta-analytic review. *Behav. Brain Sci.* 35 121–143. 10.1017/S0140525X11000446 22617651 PMC4329228

[B36] Matsumoto D. (1993). Ethnic differences in affect intensity, emotion judgments, display rule attitudes, and self-reported emotional expression in an american sample. *Motiv. Emot.* 17 107–123. 10.1007/BF00995188

[B37] Matsumoto D. Yoo S. H. Nakagawa S. (2008). Culture, emotion regulation, and adjustment. *J. Pers. Soc. Psychol.* 94 925–937. 10.1037/0022-3514.94.6.925 18505309

[B38] Mayer J. D. Gaschke Y. N. (1988). The experience and meta-experience of mood. *J. Pers. Soc. Psychol.* 55 102–111. 10.1037/0022-3514.55.1.102 3418484

[B39] Muthén L. K. Muthén B. O. (1998/2015). *Mplus User’s Guide: Statistical Analysis with Latent Variables*, 7th Edn. Los Angeles, CA: Muthén & Muthén.

[B40] Nezlek J. B. Kuppens P. (2008). Regulating positive and negative emotions in daily life. *J. Pers.* 76 561–580. 10.1111/j.1467-6494.2008.00496.x 18399953

[B41] Öhman A. (1993). “Fear and anxiety as emotional phenomena: clinical phenomenology, evolutionary perspectives and information processing mechanisms,” in *Handbook of Emotion*, eds Lewis M. Haviland J. M. (New York, NY: The Guilford Press), 511–536.

[B42] Papousek I. Freudenthaler H. H. Schulter G. (2008). The interplay of perceiving and regulating emotions in becoming infected with positive and negative moods. *Pers. Individ. Dif.* 45 463–467. 10.1016/j.paid.2008.05.021

[B43] Papousek I. Freudenthaler H. H. Schulter G. (2011). Typical performance measures of emotion regulation and emotion perception and frontal EEG asymmetry in an emotional contagion paradigm. *Pers. Individ. Dif.* 51 1018–1022. 10.1016/j.paid.2011.08.013

[B44] Preacher K. J. Hayes A. F. (2004). SPSS and SAS procedures for estimating indirect effects in simple mediation models. *Behav. Res. Methods* 36 717–731. 10.3758/BF03206553 15641418

[B45] Prochazkova E. Kret M. E. (2017). Connecting minds and sharing emotions through mimicry: a neurocognitive model of emotional contagion. *Neurosci. Biobehav. Rev.* 80 99–114. 10.1016/j.neubiorev.2017.05.013 28506927

[B46] Pugh S. D. (2001). Service with a smile: emotional contagion in the service encounter. *Acad. Manage. J.* 44 1018–1027.

[B47] Roberts N. A. Levenson R. W. Gross J. J. (2008). Cardiovascular costs of emotion suppression cross ethnic lines. *Int. J. Psychophysiol.* 70 82–87. 10.1016/j.ijpsycho.2008.06.003 18621086 PMC2583175

[B48] Scarantino A. (2012). How to define emotions scientifically. *Emot. Rev.* 4 358–368. 10.1177/1754073912445810

[B49] Schoenewolf G. (1990). Emotional contagion: behavioral induction in individuals and groups. *Mod. Psychoanal.* 15 49–61.

[B50] Serra Poirier C. Brendgen M. Vitaro F. Dionne G. Boivin M. (2017). Contagion of anxiety symptoms among adolescent siblings: a twin study. *J. Res Adol.* 27 65–77. 10.1111/jora.12254 28498537

[B51] Solomon R. L. Corbit J. D. (1978). An opponent-process theory of motivation. *Am. Econ. Rev.* 68 12–24.

[B52] Sonnby-Borgström M. Jönsson P. Svensson O. (2008). Gender differences in facial imitation and verbally reported emotional contagion from spontaneous to emotionally regulated processing levels. *Scand. J. Psychol.* 49 111–122. 10.1111/j.1467-9450.2008.00626.x 18352980

[B53] Tugade M. M. Fredrickson B. L. (2007). Regulation of positive emotions: emotion regulation strategies that promote resilience. *J. Happiness Stud.* 8 311–333. 10.1007/s10902-006-9015-4

[B54] Vaish A. Grossmann T. Woodward A. (2008). Not all emotions are created equal: the negativity bias in social-emotional development. *Psychol. Bull.* 134 383–403. 10.1037/0033-2909.134.3.383 18444702 PMC3652533

[B55] Volante M. Babu S. V. Chaturvedi H. Newsome N. Ebrahimi E. Roy T. (2016). Effects of virtual human appearance fidelity on emotional contagion in affective inter-personal simulations. 2 IEEE Trans. *Vis. Comput. Graph.* 2 1326–1335. 10.1109/TVCG.2016.2518158 26780808

[B56] Wang Y. Wang Z. H. Qiu S. S. (2013). The reliability and validity of chinese version emotional contagion scale among college students. *J. Chin. Ment. Health* 27 59–63.

[B57] Waters S. F. West T. V. Karnilowicz H. R. Mendes W. B. (2017). Affect contagion between mothers and infants: examining valence and touch. *J. Exp. Psychol. Gen.* 146 1043–1051. 10.1037/xge0000322 28493755 PMC5499532

[B58] Wegener D. T. Petty R. E. (1994). Mood management across affective states: the hedonic contingency hypothesis. *J. Pers. Soc. Psychol.* 66 1034–1048. 10.1037/0022-3514.66.6.1034 8046576

[B59] Wu Y. Babu S. V. Armstrong R. Bertrand J. W. Luo J. Roy T. (2014). Effects of virtual human animation on emotional contagion in simulated inter-personal experiences. *IEEE Trans. Vis. Comput. Graph.* 20 626–635. 10.1109/TVCG.2014.19 24650990

[B60] Xiong X. Y. (1994). *Ethnic Psychology and Ethnic Consciousness.* Kunming: University of Yunnan Press.

[B61] Zhang J. J. (2010). *New Views of Linguistic cognitive—A Dialectic Discussion.* Beijing: Higher Education Press.

[B62] Zhong J. Qian M. Y. (2005). The reliability and validity of Chinese Mood Adjective Check List. *J. Chin. Clin. Psychol.* 13 9–13.

[B63] Zhou T. Q. (2007). Emotion concern: the way of social harmony in the perspective of confucian ethics. *J. Henan Normal University* 1:40.

[B64] Zhu Y. (2015). The role of qing, (positive emotions) and li (rationality) in Chinese entrepreneurial decision making: a Confucian ren - yi, wisdom perspective. *J. Bus. Ethics* 126 613–630. 10.1007/s10551-013-1970-1

[B65] Zimmermann P. Iwanski A. (2014). Emotion regulation from early adolescence to emerging adulthood and middle adulthood: age differences, gender differences, and emotion-specific developmental variations. *Int. J. Behav. Dev.* 38 182–194. 10.1177/0165025413515405

